# How Many Patients with Choroidal Melanoma Would Be Eligible for Neoadjuvant Systemic Therapy to Enable Ruthenium-106 Brachytherapy?

**DOI:** 10.3390/cancers17183022

**Published:** 2025-09-16

**Authors:** Bertil Damato, Antonio Eleuteri, Sarah E. Coupland, Helen Kalirai, Heinrich Heimann

**Affiliations:** 1 Ocular Oncology Service, Moorfields Eye Hospital, London EC1V 2PD, UK; 2 Department of Eye and Vision Science, Institute of Life Course and Medical Sciences University of Liverpool, Liverpool L7 8TX, UK; s.e.coupland@liverpool.ac.uk (S.E.C.); h.kalirai@liverpool.ac.uk (H.K.); 3 Research & Innovation Department, Royal Liverpool University Hospital, NHS University Hospitals of Liverpool Group (UHLG), Liverpool L7 8XP, UK; antonio.eleuteri@liverpoolft.nhs.uk; 4 Liverpool Ocular Oncology Service, NHS University Hospitals of Liverpool Group (UHLG), Liverpool L7 8YE, UK

**Keywords:** uveal melanoma, neoadjuvant therapy, brachytherapy, vision, monosomy 3, metastasis

## Abstract

Background/Objectives: Neoadjuvant systemic therapy may enhance ocular and visual preservation, also prolonging life in patients with choroidal melanoma. We investigated how many choroidal melanomas would be eligible for such treatment to enable Ruthenium-106 brachytherapy. Methods: The cohort comprised 5859 patients treated for choroidal melanoma at the Liverpool Ocular Oncology Centre between 1993 and 2023. Results: If the objective is ocular conservation, then, after excluding tumors > 16 mm in diameter, involving disc and/or more than two clock hours of angle or iris, and/or extending extraocularly, approximately 60.5%, 65.1%, and 67.6% of patients would remain eligible for neoadjuvant systemic therapy, according to whether the maximum allowable tumor thickness is 8 mm, 10 mm or 12 mm, respectively. If the objective is preservation of 20/80 vision, and if exclusion criteria also include vision worse than 20/80 and tumor extension to within 3 mm of optic disc and/or fovea, then 31.0%, 33.2% and 34.1% of tumors would remain in the three tumor-thickness groups, respectively. Chromosome 3 loss would be found in approximately 33%, 52% and 56% of tumors measuring 11–12 mm, 13–14 mm and >14 mm, respectively. Conclusions: Based on the provided data and with effective neoadjuvant treatment, approximately two thirds of subjects with choroidal melanoma requiring enucleation could potentially become candidates for ruthenium-106 brachytherapy and as many as one third could also have the potential for preservation of useful vision.

## Simple Summary

In most European centers, the first choice of treatment for choroidal melanoma is Ruthenium-106 brachytherapy, then administering other forms of radiotherapy if these are available, and resorting to enucleation if these are not possible or if they are unlikely to conserve a useful and comfortable eye. Despite successful ocular treatment, about 50% of patients develop systemic metastases, which arise almost exclusively from tumors showing chromosome 3 loss. Neoadjuvant systemic therapy may avoid the need for enucleation and also delay or prevent metastatic disease and death. With data from 5859 patients, we estimate that with effective neoadjuvant treatment, approximately two thirds of patients could become candidates for ruthenium-106 brachytherapy and one third could also have the potential for preservation of useful vision. Depending on tumor height and diameter, 33% to 56% of tumors could show chromosome-3 loss, so the systemic adjuvant treatment may prolong life in these patients.

## 1. Introduction

Treatment for choroidal melanoma is aimed at preventing metastatic disease, if possible, and also conserving the eye and useful vision [1]. In most centers across Europe, the first choice of treatment is brachytherapy administered with a Ruthenium-106 applicator [2]. This emits beta radiation, which has a short range so that it is recommended only for tumors not exceeding 5 mm in thickness. Tumors with a greater height are therefore treated with iodine-125 brachytherapy, proton beam radiotherapy, or stereotactic radiotherapy [3,4,5]. If these are not available and if the patient cannot receive such treatment elsewhere, then enucleation may be required. Even if these therapeutic modalities are available, the patient may undergo enucleation because the large size of their tumor threatens severe radiation-induced exudative retinal detachment and painful neovascular glaucoma (i.e., ‘toxic tumor syndrome’) [6]. This condition may in some patients be prevented or treated by methods such as anti-angiogenic therapy, laser therapy, endoresection and exoresection [6,7]; however, larger tumor size also requires a greater radiation dose and a larger radiation volume so that there is a higher risk of irreversible visual loss from radiation-induced optic neuropathy and/or maculopathy. Greater tumor size also indicates an increased likelihood that subclinical micrometastases are already present, eventually proving fatal unless death occurs from unrelated disease [8]. For these reasons, there is scope for neoadjuvant systemic therapy for patients with advanced choroidal melanoma to shrink the tumor so that Ruthenium-106 brachytherapy is possible, also reducing the risk of visual loss from toxic tumor syndrome and/or collateral damage to optic nerve, fovea or both, possibly suppressing any micrometastases that may be present.

Ruthenium-106 brachytherapy has several contraindications other than thickness exceeding 5 mm. First, this treatment is possible only for tumors not exceeding 16 mm in basal diameter if a 2 mm safety margin is administered in accordance with accepted guidelines, unless an applicator larger than 20 mm is used [9]. Second, optic disc involvement by the tumor is associated with an increased rate of local treatment failure, because the optic nerve physically prevents ideal plaque placement, so that an adequate safety margin is not possible [10]. Thirdly, circumferential spread around the angle may be more extensive than is apparent on gonioscopy so that we advocate a lateral safety margin of one clock hour at the limbus to prevent local treatment failure [11]. We therefore consider tumor involvement of more than two clock hours of angle to be a contraindication as this would therefore require treatment of at least four clock hours of angle, with the anterior plaque edge located 4 mm anterior to the limbus. Fourthly, extraocular tumor extension increases the risk of orbital recurrence and, if bulky, prevents proper positioning of the plaque against the sclera.

If the objective of treatment is to conserve not only the eye but also useful vision, then additional contraindications to Ruthenium-106 brachytherapy may include tumor extension to within two disc-diameters (DD) of the optic disc and/or fovea, and conditions irreversibly reducing the initial visual acuity [12,13,14].

The aim of the present investigation was to estimate the proportion of patients with choroidal melanoma who would be eligible for neoadjuvant systemic therapy, such as Darovasertib, aimed at enabling Ruthenium-106 brachytherapy. Another aim was to determine the proportion of patients having a tumor with chromosome 3 loss (i.e., monosomy 3), and hence a significant risk of metastatic disease, which may be prevented or delayed by the systemic adjuvant therapy.

## 2. Materials and Methods

The study sample consisted of a consecutive series of 5859 adult patients who were treated for choroidal melanoma at the Liverpool Ocular Oncology Centre between 1993 and 2023.

The diagnosis of melanoma was based on generally accepted clinical features, in some cases confirming malignancy by documenting tumor growth or by biopsy [15]. Treatment was selected according to tumor size, location, and extent, taking account of the patient’s preferences. Our first choice was Ruthenium-106 brachytherapy if the tumor thickness did not exceed 5 mm and if we were confident that the plaque could accurately positioned over the tumor. Minimum radiation doses of 350 Gy and 100 Gy were routinely prescribed to sclera and tumor apex, respectively, and plaque placement over the tumor was confirmed intraoperatively by performing transillumination through perforations in a plastic template designed by the first author (Damato Ruthenium plaque template, Altomed, Boldon, UK) while performing binocular indirect ophthalmoscopy (i.e., ‘sunrise procedure’) [12]. If the tumor was too large for Ruthenium plaque brachytherapy or if the plaque could not be accurately positioned over the tumor, then proton beam radiotherapy, endoresection or exoresection was preferred. Enucleation tended to be undertaken if these therapeutic modalities were not possible or if the patient was not motivated to retain the eye.

Visual acuity was measured with LOGMar charts by us and by a variety of methods at the patient’s local hospital. Tumor dimensions were measured with b-scan ultrasonography, determining tumor thickness by placing the calipers at the internal scleral surface and at the tumor apex, excluding retina. Distances from posterior tumor margin to optic disc and fovea were determined ophthalmoscopically or by assessment of fundus photographs, with tumor-to-disc distances measured by ultrasonography in patients undergoing proton beam radiotherapy.

From 1999 onwards, patients were offered genetic tumor analysis if they underwent enucleation, and, from 2007 onwards, those treated with radiotherapy were offered prognostic tumor biopsy [15,16]. Genetic tumor analysis of the uveal melanoma was performed with fluorescence in situ hybridization (FISH) until around 2007 and then with multiplex ligation-dependent probe amplification (MLPA) or, if the tumor sample was small, microsatellite analysis (MSA) [17,18,19].

Clinical data were collected prospectively, using paper forms until around 2015 and then with electronic forms, both of which minimized free text. A data manager then entered this information on a computerized database (Version 9.4, Revelation Software, Westwood, NJ, USA), which was customized for our ocular oncology service by Sprezzatura (Sprezzatura Ltd., London, UK). Statistical analyses were performed with Stata/IC (Version 16.1, StataCorp, College Station, TX, USA).

The following baseline data were analyzed: age, sex, affected eye, visual acuity in affected eye, largest basal tumor diameter, tumor thickness, location of posterior tumor margin with respect to optic disc and/or fovea, location of anterior tumor margin, extraocular tumor extension, type of ocular treatment, tumor cell type, mitotic count, extravascular matrix pattern, chromosome 3 status, and chromosome 8q status.

Our exclusion criteria for Ruthenium brachytherapy with a 20 mm applicator were: largest basal tumor diameter exceeding 16.4 mm, extraocular tumor spread, optic disc involvement, and involvement of more than two clock hours of iris and/or angle. Relative exclusion criteria included: tumor height exceeding 8 mm, 10 mm or 12 mm; minimum tumor height less than 4 mm or 6 mm; tumor extension to within two disc diameters of the optic disc; and initial visual acuity worse than 20/40, 20/80, or 20/200.

We estimated the confidence intervals using the Wilson score method [20]. This provides a good approximation even with small observed proportions (this is particularly relevant for the entries in Table 1).

With ethical committee approval, from the early 1990s on arrival at our center, all patients were asked to sign a form providing consent for the use of their data, tissues and images to be used for research, teaching, and audit. The present study was approved by the Audit Facilitator at the Royal Liverpool University Hospital (Audit code: Ophth/CA/2025/26/07). In view of its hypothetical nature, not influencing care, specific patient consent for this study was not required. We conducted our investigations in accordance with the Declaration of Helsinki.

## 3. Results

### 3.1. Proportion of Cases Eligible for Neoadjuvant Therapy to Conserve the Globe with Ruthenium-106 Brachytherapy

After excluding tumors more than 16 mm in basal diameter, involving optic disc or more than two clock hours of angle or iris, and/or extending extraocularly, approximately 60.5%, 65.1% and 67.6% of patients would be eligible for neoadjuvant systemic therapy, according to whether the maximum tumor thickness is 8 mm, 10 mm or 12 mm, respectively (Table 1).

### 3.2. Proportion of Cases Eligible for Neoadjuvant Therapy to Conserve Visual Acuity with Ruthenium-106 Brachytherapy

After excluding tumors listed in Table 1, if further cases are excluded because the tumor thickness is 3 mm or less, then there would be 25.4%, 30.0%, and 32.5% of tumors remaining with a tumor height of 4–8 mm, 4–10 mm and 4–12 mm, respectively (Table 2). If additional tumors are excluded because of visual loss at presentation, then there would be fewer patients remaining (e.g., 22.9% in patients with a tumor height of 4–10 mm and visual acuity worse than 20/80 at presentation). If additional patients are excluded because the tumor extends within 2 DD of optic disc or fovea, there would be even fewer patients remaining (e.g., 19.3% in patients with a tumor height of 4–10 mm, reducing to 15.8% if the presenting visual acuity is worse than 20/80) (Table 2).

If the minimum tumor thickness is 6 mm, then fewer patients would be eligible (e.g., 11% if the maximum tumor height is 10 mm and if the tumor extends within 2 DD of the optic disc or fovea, reducing further to 8% if the visual acuity at presentation is worse than 20/80) (Table 3).

### 3.3. Prevalence of Chromosome 3 Loss According to Basal Tumor Diameter and Tumor Height

The prevalence of chromosome 3 loss would range approximately from 31% in tumors measuring 11–12 mm in diameter and up to 8 mm in thickness to 56% in tumors measuring 14–16 mm in diameter and up to 12 mm in thickness (Table 4).

## 4. Discussion

### 4.1. Main Findings

This study indicates the proportions of patients who would be eligible for systemic neoadjuvant therapy aimed at enabling Ruthenium-106 brachytherapy instead of enucleation and aimed at improving vision outcomes. We also report the proportions of patients with a high risk of metastasis, whose life may perhaps be prolonged by this systemic therapy. These data may be useful in the planning of clinical trials evaluating this neoadjuvant therapy.

### 4.2. Methods

Our eligibility criteria for Ruthenium-106 brachytherapy that are defined in this study require justification.

Optic disc involvement was considered an exclusion criterion because it is associated with a high risk of local treatment failure. Involvement of more than two clock hours of angle or iris involvement was considered the limit for Ruthenium brachytherapy because we administer the radiotherapy with a one-clock hour safety margin on each side of the tumor.

The 16 mm basal diameter limit is in keeping with current guidelines regarding 2 mm safety margins if a 20 mm Ruthenium-106 applicator is used; however, our experience with adjunctive brachytherapy after trans-scleral exoresection of large choroidal melanomas suggests that wider safety margins are needed except perhaps if a high dose scleral dose of radiation is administered (e.g., minimum 350 Gy to sclera), which increases radiation side scatter beyond the plaque limits.

Extraocular tumor spread is generally regarded as a contraindication to brachytherapy because bulky extraocular tumor may tilt the plaque away from the sclera, preventing adequate irradiation of the intraocular tumor; however, this may not be a problem with tumors showing minimal extraocular spread.

When the objective of brachytherapy is to conserve vision, tumor extension to within 2 DD of the optic disc and/or fovea is associated with an increased risk of collateral radiation damage to these structures. This is because it likely that the full dose of radiation will be administered to the area of choroid involved by the tumor before the neoadjuvant therapy, together with an additional 2–3 mm safety margin surrounding the margins of the untreated tumor. Similarly, poor pre-treatment visual acuity reduces the chances of preserving vision, often because of involvement of the fovea by tumor or retinal detachment.

We reported patient eligibility according to a range of minimum tumor thicknesses because some may not consider neoadjuvant therapy to be indicated if the tumor at presentation is already small enough for Ruthenium-106 brachytherapy whereas others may wish to administer neoadjuvant therapy even if the tumor thickness does not exceed 5 mm, to reduce the risk of visual loss from collateral radiation-induced damage to optic nerve and/or fovea and from maculopathy caused by fluid or lipid exuding from the irradiated tumor (i.e., ‘toxic tumor syndrome’) [6].

We analyzed tumors with maximum allowable thicknesses of 8 mm, 10 mm and 12 mm separately because the chances of the tumor shrinking enough for Ruthenium-106 brachytherapy will vary according to the therapeutic agents used for the neoadjuvant treatment and perhaps other factors, such as tumor shape, mitotic count, and genetic makeup. Some authors have reported good outcomes after Ruthenium-106 brachytherapy for tumors more than 5 mm thick [21]. After neoadjuvant therapy, such brachytherapy may be successful even if the tumor thickness does not diminish to less than 6 mm.

In view of the possibility that neoadjuvant systemic therapy would suppress systemic micrometastases to prolong life, we report the prevalence of monosomy 3, which is well known to be associated with an increased risk of metastatic death. For example, according to the Liverpool Uveal Melanoma Prognosticator Online (LUMPO), (www.LUMPO.net, accessed 13 September 2025), a 60-year-old patient with a choroidal melanoma measuring 16 mm in diameter and 8 mm in thickness would have about a 54% risk of chromosome 3 loss and, therefore, about a 45% risk of metastatic death within 10 years (because chromosome 3 loss is usually associated with epithelioid melanoma cytomorphology, high mitotic count, closed loops, and chromosome 8q gain, and, therefore, a 10-year metastatic mortality of approximately 83%). Survival probability of individual patients with different combinations of metastasis predictors can be determined using prognostic tools such as the LUMPO and by referring to other published studies [22,23]. The potential benefit of neoadjuvant systemic therapy in patients with a high risk of metastatic disease has yet to be determined.

### 4.3. Strengths and Weaknesses

To our knowledge, there are no published studies estimating number of patients who would be eligible for neoadjuvant systemic therapy aimed at conserving the eye and vision with Ruthenium-106 brachytherapy or, indeed, other forms of radiotherapy.

The main strength of the study is the large number of tumors, many of which have been genetically typed.

Our study has several limitations. First, in the early years, genetic tumor analysis was performed with FISH, which is less sensitive than current methods. Since then, several genetic metastasis predictors have been discovered [24]. Consequently, the rate of metastatic death in patients with reported disomy 3 may prove to be higher with current methods, such as next generation sequencing [25].

Second, we did not take account of limited life expectancy, occurring because of old age and/or serious illness, such as cancer and cardiovascular disease. We also did not consider diabetes as an exclusion factor. These conditions may be considered exclusion criteria in clinical trials but may not prevent neoadjuvant therapy of patients in routine clinical practice, especially if the vision in the fellow eye is poor.

Third, we did not exclude patients with diffuse choroidal melanoma, which is rare, and did not consider glaucoma and other co-morbidities as exclusion criteria.

Although we excluded all tumors with optic disc involvement, some of these tumors can be successfully treated if the disc involvement is minimal and if a high radiation dose is administered.

Similarly, extraocular tumor spread may not exclude plaque radiotherapy if it is small or if it can be surgically excised because it is located far anteriorly, over ciliary body [26].

In clinical practice, the decision whether to attempt systemic neoadjuvant therapy will be influenced by the cost of this treatment, likely side effects, and other factors not considered in the present study.

### 4.4. Further Studies

It would be ideal if the impact of neoadjuvant therapy on Ruthenium-106 radiotherapy could be studied at other centers, where the indications for this radiotherapy may be different from ours, where patient cohorts may show different prevalences of the eligibility criteria, and where methods for measuring tumor dimensions, visual acuity, and other factors may be different.

There is also scope for similar studies evaluating systemic neoadjuvant therapy prior to other forms of radiotherapy, such as Iodine-125 brachytherapy, proton beam radiotherapy, and stereotactic radiotherapy, which are more likely to cause toxic tumor syndrome and collateral damage to optic nerve and/or fovea when administered for large tumors. The impact of neoadjuvant therapy on laser therapy, endoresection and trans-scleral exoresection may also be worth investigating in due course.

There is a need to evaluate 3-D dosimetry programs for predicting reduction in radiation doses to optic nerve and fovea when the objective of systemic neoadjuvant therapy is to conserve vision [27,28].

It would be useful to determine the impact of anti-angiogenic therapy on visual outcomes, whether such treatment is administered prophylactically or when macular edema and other features of toxic tumor syndrome have developed [29,30].

With brachytherapy, the outcome of neoadjuvant therapy will depend greatly on the accuracy of plaque placement in relation to the tumor so that it will be important to document measures that are taken to achieve this accuracy, such as intraoperative ultrasonography, trans-scleral transillumination through perforations in the plaque template, and documentation of plaque-to-limbus distance [12].

Ideally, genetic tumor typing would be performed when including patients into any clinical trials investigating the impact of systemic neoadjuvant or adjuvant therapy on survival. Genetic analysis may also reveal predictors of tumor response to the neoadjuvant systemic therapy.

There is scope for studies to inform the management of patients who undergo tumor biopsy when considering neoadjuvant therapy and who are found to have genetic aberrations indicating a high risk of metastatic disease. The question arises as to whether such patients should receive stronger treatment, such as Darovasertib/Crizotinib and Tebentafusp despite the increased risk of side effects [31,32].

## 5. Conclusions

It is likely that systemic adjuvant therapy for choroidal melanoma prior to ocular radiotherapy will become more widespread as new therapeutic agents are developed and as the risks and benefits of this approach become known. This study suggests that if the objective of such treatment is to conserve the eye with Ruthenium-106 brachytherapy, then approximately two thirds of patients with choroidal melanoma would be considered eligible. This proportion would diminish to as little as 7% with an increasing number of exclusion criteria, such as tumor thickness less than 4 mm or 6 mm; tumor extension to within 2 DD of optic disc and/or fovea, and/or visual acuity worse than 20/200, 20/80 or 20/40. Chromosome 3 loss and, therefore, other metastasis predictors, would be found in approximately 33–56% of tumors, depending on tumor diameter. These data should be useful when designing clinical trials and to ophthalmologists considering inclusion of their patients in such trials.

## Figures and Tables

**Table 1:** Proportion of patients eligible for neoadjuvant systemic therapy if administered to enable Ruthenium plaque radiotherapy for choroidal melanoma to conserve the eye. The 95% confidence intervals were estimated using the Wilson approximation.

Exclusion Criteria	Maximum Tumor Height = 8 mm	Maximum Tumor Height = 10 mm	Maximum Tumor Height = 12 mm
	Percent remaining (95% CI)	Percent remaining (95% CI)	Percent remaining (95% CI)
Height > maximum limit	81.8% (80.8%, 82.8%)	91.0% (90.3%, 91.7%)	96.5% (96.0%, 97.0%)
Extraocular spread	79.5% (78.5%, 80.5%)	88.0% (87.1%, 88.8%)	93.0% (92.3%, 93.6%)
Disc involved	66.6% (65.4%, 67.8%)	74.2% (73.1%, 75.3%)	78.8% (77.7%, 79.8%)
Iris/Angle involvement > 2 clock hrs	65.3% (64.0%, 66.5%)	72.4% (71.2%, 73.5%)	76.5% (75.4%, 77.6%)
Diameter > 16 mm	60.5% (59.2%, 61.7%)	65.1% (63.9%, 66.3%)	67.6% (66.4%, 68.8%)

CI Confidence Interval.

**Table 2:** Proportion of patients eligible for neoadjuvant systemic therapy if administered to enable Ruthenium plaque radiotherapy for choroidal melanoma to preserve vision if the minimum tumor height is 4 mm. The 95% confidence intervals were estimated using the Wilson approximation.

Exclusion Criteria	Maximum Tumor Height = 8 mm	Maximum Tumor Height = 10 mm	Maximum Tumor Height = 12 mm
	Percent remaining (95% CI)	Percent remaining (95% CI)	Percent remaining (95% CI)
Thickness ≤ 3 mm	25.4% (24.3%, 26.5%)	30.0% (28.9%, 31.2%)	32.5% (31.3%, 33.7%)
Initial vision worse than 20/200	22.8% (21.8%, 23.9%)	26.2% (25.1%, 27.4%)	27.9% (26.7%, 29.0%)
Initial vision worse than 20/80	20.4% (19.4%, 21.5%)	22.9% (21.9%, 24.0%)	24.0% (22.9%, 25.1%)
Initial vision worse than 20/40	16.7% (15.8%, 17.7%)	18.5% (17.5%, 19.5%)	19.1% (18.1%, 20.2%)
Tumor within 2DD of disc or fovea	15.6% (14.7%, 16.6%)	19.3% (18.3%, 20.4%)	21.4% (20.4%, 22.5%)
Initial vision worse than 20/200	14.6% (13.7%, 15.5%)	17.4% (16.4%, 18.3%)	18.8% (17.8%, 19.8%)
Initial vision worse than 20/80	13.6% (12.7%, 14.5%)	15.8% (14.8%, 16.7%)	16.7% (15.7%, 17.6%)
Initial vision worse than 20/40	11.8% (11.0%, 12.7%)	13.3% (12.5%, 14.2%)	13.9% (13.0%, 14.8%)

CI, Confidence Interval; DD, Disc Diameter (1DD = 1.5 mm).

**Table 3:** Proportion of patients eligible for neoadjuvant systemic therapy if administered to enable Ruthenium plaque radiotherapy for choroidal melanoma to preserve vision if the minimum tumor height is 6 mm. The 95% confidence intervals were estimated using the Wilson approximation.

Exclusion Criteria	Maximum Tumor Height = 8 mm	Maximum Tumor Height = 10 mm	Maximum Tumor Height = 12 mm
	Percent remaining (95% CI)	Percent remaining (95% CI)	Percent remaining (95% CI)
Thickness ≤ 5 mm	10.9% (10.2%, 11.7%)	15.6% (14.7%, 16.6%)	18.0% (17.1%, 19.0%)
Initial vision worse than 20/200	9.2% (8.5%, 10.0%)	12.6% (11.8%, 13.5%)	14.3% (13.4%, 15.2%)
Initial vision worse than 20/80	8.0% (7.3%, 8.7%)	10.5% (9.7%, 11.3%)	11.6% (10.8%, 12.4%)
Initial vision worse than 20/40	6.2% (5.6%, 6.8%)	8.0% (7.3%, 8.7%)	8.6% (7.9%, 9.3%)
Tumor within 2DD of disc or fovea	7.3% (6.6%, 8.0%)	11.0% (10.2%, 11.8%)	13.1% (12.2%, 13.9%)
Initial vision worse than 20/200	6.5% (5.9%, 7.2%)	9.3% (8.6%, 10.1%)	10.7% (10.0%, 11.5%)
Initial vision worse than 20/80	5.9% (5.3%, 6.5%)	8.0% (7.4%, 8.7%)	8.9% (8.2%, 9.7%)
Initial vision worse than 20/40	4.8% (4.2%, 5.3%)	6.3% (5.7%, 6.9%)	6.8% (6.2%, 7.5%)

CI, Confidence Interval; DD, Disc Diameter (1DD = 1.5 mm).

**Table 4:** Prevalence of monosomy 3 according to basal tumor diameter and maximum tumor thickness in patients eligible for neoadjuvant therapy to conserve the eye. The 95% confidence intervals have been estimated using the Wilson approximation.

Max. Tumor Ht. (mm)	Largest Basal Diameter (mm)	Chromosome 3 *	
		Disomy (95% CI)	Monosomy (95% CI)
8	11–12	69% (62%, 74%)	31% (26%, 38%)
	13–14	50% (44%, 57%)	50% (43%, 56%)
	15–16	46% (39%, 53%)	54% (47%, 61%)
10	11–12	68% (62%, 73%)	32% (27%, 38%)
	13–14	49% (43%, 55%)	51% (45%, 57%)
	15–16	44% (38%, 51%)	56% (49%, 62%)
12	11–12	67% (61%, 72%)	33% (28%, 39%)
	13–14	48% (42%, 53%)	52% (47%, 58%)
	15–16	44% (38%, 50%)	56% (50%, 62%)

CI, Confidence Interval; * Minimum Tumor Ht 4 mm.

## Data Availability

No new data were created.

## Abbreviations

The following abbreviations are used in this manuscript:

CI	Confidence Interval
DDs	Disc Diameters
FISH	Fluorescence In Situ Hybridization
Gy	Grays
LUMPO	Liverpool Uveal Melanoma Prognosticator Online
MSA	Microsatellite Analysis
MLPA	Multiplex Ligation-dependent Probe Amplification
